# New Plant Extracts Exert Complementary Anti‐Hair Loss Properties in Human In Vitro and Ex Vivo Models

**DOI:** 10.1111/jocd.16616

**Published:** 2024-11-28

**Authors:** Daniel Bacqueville, Marguerite Lévêque, Camille Mas, Marie‐José Haure, Anaïs Noustens, Valérie Mengeaud, Sophie Carrère, Sandrine Bessou‐Touya, Hélène Duplan, Nathalie Castex Rizzi, Jean‐Hilaire Saurat

**Affiliations:** ^1^ R & D Department Pierre Fabre Dermo‐Cosmétique and Personal Care Toulouse France; ^2^ Medical Department Laboratoires Dermalogiques DUCRAY Lavaur France; ^3^ University of Geneva Geneva Switzerland

**Keywords:** active ingredients, anchorage, dermal papilla, hair growth, hair loss, human hair follicle

## Abstract

**Background:**

Hair loss is linked to dysfunction of the growth (anagen), regression (catagen) and rest (telogen) phases of the hair follicle (HF) cycle.

**Aims:**

To evaluate the effects of a *Silybum marianum* extract (SME), manganese PCA (MnPCA), and a *Lespedeza capitata* extract (LCE) on markers of hair growth and anchorage in human follicle dermal papilla cells (HFDPCs), and to investigate the ability of a topical serum containing these active ingredients to improve HF growth in an *ex vivo* human scalp skin model.

**Methods:**

In HFDPCs, we assessed receptor tyrosine kinase phosphorylation and Wnt/β‐catenin pathway activation; quantified versican, vascular endothelial growth factor (VEGF) and Dickkopf‐1 (DDK1) secretion; and evaluated 5α‐reductase (5αR) activity. Using scalp skin biopsies from two female donors, we measured hair shaft elongation, analyzed hair matrix keratinocyte proliferation and apoptosis, and determined HF cycle stage and score.

**Results:**

Compared to untreated HFDPCs, SME upregulated phosphorylation of growth factor receptors (EGFR:1.9 × and PDGFR: 2.8 ×) and their downstream effectors (ERK, GSK3, Akt, and STAT: 1.2–2.0 ×); MnPCA enhanced versican (33.0 ×) and VEGF (3.3 ×) secretion, and stimulated the Wnt/β‐catenin pathway (+80%); and LCE reduced DKK1 secretion (−72%) and 5αR activity (dihydrotestosterone/testosterone ratio: −60%). Compared to untreated scalp skin biopsies, the serum enhanced hair shaft elongation (+102%), and significantly prolonged the anagen phase by improving hair cycle scores and stimulating hair matrix keratinocyte proliferation (+58%).

**Conclusions:**

SME, MnPCA, and LCE displayed complementary anti‐hair loss properties. The serum combining these active ingredients may be useful in hair loss treatment.

## 1 Introduction

Alopecia (hair loss) has a major impact on quality of life and self‐esteem in both men and women [1] and is therefore a common reason for dermatological consultation. Topical minoxidil and oral finasteride, which are currently the standard treatments for female and male pattern hair loss, are not effective in all patients and can cause side effects in some cases [2, 3]. Advances in our understanding of the molecular mechanisms of hair morphogenesis and biology, as well as of the pathological basis of alopecia [4, 5, 6], have paved the way for the development of new therapeutic candidates for alopecia treatment.

Hair growth is a continuous process characterized by cycles of growth (anagen), apoptosis‐mediated regression (catagen), and rest (telogen) [4, 7, 8]. These cycles are tightly regulated by numerous molecular interactions occurring within the hair follicles (HFs), which are “mini‐organs” composed of different compartments including dermal papilla cells, stem cells residing in the bulge region of the outer root sheath, and HF matrix keratinocytes [4, 7, 8]. These processes are fine‐tuned by specific intrafollicle and extra‐follicle signals (e.g., growth factors, cytokines, hormones, and enzymes) that regulate downstream signaling pathways. In dermal papilla cells, the Wnt/β‐catenin signaling pathway plays a key role in stimulating hair growth [9, 10]. Upregulation of the extracellular matrix protein versican [11, 12, 13], as well as of vascular endothelial growth factor (VEGF) [4] and platelet‐derived growth factor (PDGF) [14, 15, 16], has also been associated with the anagen phase. Androgens such as dihydrotestosterone (DHT)—the active metabolite of testosterone produced through the action of the enzyme 5‐alpha‐reductase (5αR)—have been shown to impair dermal papilla‐induced HF stem cell differentiation by inhibiting Wnt signaling in androgenetic alopecia [17, 18, 19]. Moreover, Dickkopf‐1 (DDK1), an endogenous Wnt antagonist, has been shown to be secreted by dermal papilla cells from balding patients in response to DHT [20] and to promote catagen progression [21].

A major goal of the cosmetics industry remains the identification and characterization of innovative active ingredients, especially medicinal herbs or natural/phytochemical compounds, which could specifically target these signaling pathways [22, 23, 24, 25]. The present study focused on three natural ingredients that could be of interest for treating hair loss by acting on three mechanisms, that is, stopping the telogen phase, stimulating the anagen phase, and promoting anchoring: a patented *Silybum marianum* extract (SME, WO/2021/023820), containing less than 2% silymarin; a patented *Lespedeza capitata* extract (LCE, WO/2020/020791A1); and the manganese (Mn) salt of L‐pyrrolidone carboxylic acid (MnPCA). The SME has been shown to stimulate the expression of keratin 75 (K75; patent WO/2021/023820), a type II epithelial human keratin specifically expressed in the companion layer of HFs in the anagen phase, formerly called K6hf. K75 is involved in hair shaft anchorage [26, 27], and K75 deficiency has been associated with loose anagen hair syndrome, a rare genetic alopecia [28]. Pastorino et al. [29] showed that a dry ethanolic extract of *L. capitata* significantly stimulated the proliferation of human fibroblasts and keratinocytes *in vitro*. Both *S. marianum* and *L. capitata* contain flavonoids; nutritional plants containing flavonoids have been shown to have a protective role in HF disruption [30]. Manganese levels have been shown to be significantly lower in hair of men with pattern alopecia than in hair of healthy men [31] and PCA is a physiological transporter of Mn.

The aims of this study were to evaluate the individual effects of these three active ingredients in human follicle dermal papilla cells (HFDPCs) *in vitro*, and to assess the ability of a serum containing all three active ingredients to improve HF growth in an *ex vivo* human scalp skin model. The *ex vivo* effects of the topical serum were compared to those of a commercially available lotion containing 5% minoxidil.

## 2 Materials and Methods

### 2.1 Active Ingredients and Test Products

MnPCA (Mangalidone) was obtained from Solabia, France. All other test products and compounds, including SME (dissolved in dimethylsulfoxide, DMSO) and LCE, were obtained from Pierre Fabre Laboratories (France). The test product used in the *ex vivo* studies was the commercially available Ducray NEOPTIDE EXPERT Serum, containing SME, MnPCA, and LCE as the active ingredients. A commercially available lotion containing 5% minoxidil (ALOPEXY) was used as a positive control in the *ex vivo* experiments.

### 2.2 HFDPC Culture

All *in vitro* experiments were performed in the Pierre Fabre Laboratories R&D Centre (Toulouse, France) using HFDPCs isolated from different donors (supplied by PromoCell GmbH, Germany). The HFDPCs (C‐12071) were seeded in 96‐well plates or 60‐mm Petri dishes in an adapted culture medium (C‐26500) with specific complements (C‐39625), as recommended by the supplier. They were then grown at 37°C with 5% CO_2_ for 24–48 h, until they reached about 80% confluency, before addition of any of the active ingredients. The absence of any potential cytotoxicity of SME, MnPCA, and LCE was confirmed using an ATP‐based cell viability assay (ATPlite Luminescence Assay System, PerkinElmer, USA).

### 2.3 Growth Factor Receptor Signaling Pathway Analysis

HFDPCs from two donors were treated with SME (30 μg/mL) or DMSO (0.03%) as a control for 1 h before harvesting and cell lysate analysis. Growth factor receptor (GFR) signaling pathways were analyzed using two Human Proteome Profiler antibody array kits (R&D Systems, USA) to simultaneously detect the phosphorylation of 49 receptor tyrosine kinases (Proteome Profiler Human Phospho‐RTK Array Kit) and of 43 kinases and two related total proteins (Proteome Profiler Human Phospho‐Kinase Array Kit). These membrane‐based sandwich immunoassays were used according to the manufacturer's instructions. Signal analysis was performed using the Chemidoc MP imaging system with the Image Lab (Bio‐Rad, USA), and Image J software and the MicroArray Profile plugin. All assays were performed in duplicate.

### 2.4 Wnt/β‐Catenin Signaling Pathway Analysis

Activation of the Wnt/β‐catenin signaling pathway was studied using a standard gene reporter assay. Briefly, HFDPCs from three independent donors were transfected with a lentivirus expressing a luciferase gene under the control of a Wnt/β‐catenin promoter (TCF/LEF transcription factors) using the Cignal Lenti Reporter Assay (Qiagen, USA). Transfected HFDPCs were either incubated with MnPCA 0.009% for 24 h or left untreated. Luciferase expression was then measured using the Bright‐Glo Luciferase Assay System (Promega, USA), and luminescence was quantified using a CLARIOstar microplate reader (BMG LABTECH, Germany).

### 2.5 VEGF, Versican, and DKK1 Secretion

VEGF, versican, and DKK1 levels were quantified in the culture medium of treated and untreated HFDPCs using the following commercially available immunoassay kits according to the manufacturers' instructions: the Human Versican ELISA kit (Cusabio, China) and Milliplex immunoassays (Merck Millipore, USA) for VEGF and DKK1. VEGF and versican levels were quantified following incubation of HFDPCs with MnPCA (at doses from 0.004% to 0.020%) during 24 and 48 h, respectively. DKK1 production was assessed after a 24‐h incubation of HFDPCs with 0.0003% or 0.001% LCE. Tests were performed in triplicate on untreated and treated HFDPCs from each of the three donors.

### 2.6 Measurements of 5α‐Reductase Activity

The activity of 5αR was evaluated by performing [^14^C]‐testosterone‐based metabolism studies, followed by metabolite quantification using thin‐layer chromatography (TLC). Briefly, HFDPCs from two independent donors (a woman aged 59‐year‐old and a man aged 55‐year‐old) were left untreated, or were treated for 24 h with LCE (0.01%) or finasteride (10 μM; Carbosynth Limited, UK) as positive control. Chloroform/methanol extraction was then used to separate the steroid molecules from the culture supernatant. The organic phase was collected, and testosterone and metabolites were separated by TLC by using a solvent system containing dichloromethane, ethyl acetate, and methanol. Testosterone and DHT levels were estimated by densitometric analysis using the Packard Cyclone PhosphorImager (PerkinElmer) and the Fujifilm Multigauge software. Tests were performed in triplicate.

### 2.7 Scalp Skin Biopsies and Topical Treatments

*Ex vivo* experiments were performed in the Monasterium laboratory Skin & Hair Research Solutions GmbH (Muenster, Germany), using discarded temporal scalp skin samples containing terminal hair from two healthy Caucasian females (59 and 43 years old) who had undergone routine face‐lift surgery. Punch biopsies (6 mm; *n* = 6 from each donor), orientated in the direction of hair growth, were shaved with a scalpel, placed individually in 4‐cm^2^ wells, and cultured at 37°C with 5% CO_2_ in a minimal medium. After a 24 h‐resting period, the medium was changed, and the samples were either left untreated or a topical treatment (10 μL/cm^2^ of the study serum or the lotion containing 5% minoxidil) was applied daily from Day 1 to Day 4 and left for 4 h before removal of any residual product from the sample surface. The experiment was performed in duplicate for each condition.

### 2.8 Hair Shaft Elongation Measurements

Top view photos of each punch biopsy were taken daily from Day 0 to Day 5 using a digital light microscope at an original magnification of 30 × (VHX900, Keyence Corporation, Japan) and then analyzed using the affiliated software. All visible HFs were numbered and measured from the base of the epidermis to the distal part of the hair shaft. To optimize measurement accuracy, HFs growing perpendicularly to the epidermis or HFs measuring less than 130 μm on Day 1 were excluded from the analysis. Elongation was calculated as the change in hair shaft dimensions from Day 1 to Day 5.

### 2.9 Immunohistochemistry

Samples collected at Day 5 were snap frozen, embedded in cryomatrix (Thermo Fisher Scientific, UK) and stored at −80°C until further use. Sections (7 μm) were then collected using a cryostat (Leica Biosystems), mounted on slides, and stored at −80°C until immunostaining.

Proliferating and apoptotic cells in the hair matrix were stained using a Ki‐67/TUNEL (terminal dUTP nick‐end labelling) double‐labelling assay [32, 33], with a mouse anti‐Ki‐67 antibody (Cell Signaling Technology, USA) and the ApopTag Fluorescein In Situ Apoptosis Detection Kit (Merck Millipore, Germany).

Staining of K75 was performed using a guinea pig anti‐K75 antibody (Progen, Germany) and an Alexa Fluor594 goat anti‐guinea pig IgG (H + L) (Invitrogen, USA) according to the manufacturer's instructions. Counterstaining with 4′,6‐diamidino‐2‐phenylindole (DAPI, Invitrogen) was performed to visualize nuclei. Images were taken using a Nikon A1R Si confocal microscope, and immunostaining was quantified using the NIS‐Elements Nikon Imaging Software.

### 2.10 Histochemistry

Masson–Fontana staining was performed for the histochemical visualization of melanin. Cryosections were fixed in a 2:1 solution of ethanol:acetic acid and incubated with an ammonia‐based 5% silver nitrate solution in the dark at 56°C for 40 min. The sections were then incubated with 5% sodium thiosulfate solution for 1 min. Counterstaining with hematoxylin (Carl Roth GmbH & Co KG, Germany) was performed to visualize nuclei. Images were taken using a Keyence fluorescence microscope (BZ9100; Japan) using the brightfield setting.

### 2.11 HF Cycle Stage and Score

HF cycle stages were determined based on Masson–Fontana histochemistry, Ki‐67/TUNEL immunostaining, and previously defined criteria (Table S1) [34]. A standardized score [35] was applied to each HF: anagen (100), early catagen (200), mid catagen (300), late catagen (400), and dystrophic (500) HFs.

### 2.12 Hair Matrix Keratinocyte Proliferation and Apoptosis

Quantitative evaluations of proliferation and apoptosis were performed by calculating the percentage of Ki‐67+ and TUNEL+ cells relative to the total number of DAPI+ cells. Ki‐67+ nuclei were counted in the germinative hair matrix (gHM), the part of the hair matrix located below Auber's line. TUNEL+ nuclei were counted in the gHM and the precortical hair matrix (pcHM), which includes the area of the hair matrix from Auber's line to two cell lines above the dermal papilla and excludes the outer root sheath. The absence of any potential cytotoxicity of the serum was confirmed by counting ectopic melanin clumps [36] in a defined area that started at the Auber's line and including the pcHM.

### 2.13 Statistical Analyses

Data were obtained from two to four donors in experiments performed in duplicate or triplicate. *In vitro* data are presented as the mean ± standard error of the mean (SEM). Intergroup comparisons were performed using a one‐way ANOVA with a Dunnett's multiple comparison test on data normalized to the controls. *Ex vivo* data are presented as the mean ± SEM. Intergroup comparisons were performed using the unpaired *t*‐test. Statistical analyses were performed using the GraphPad Prism 9 software. Between‐group differences with *p* > 0.05 were considered statistically significant.

## 3 Results

### 3.1 Effect of *Silybum marianum* Extract on the GFR Signaling Pathway in HFDPCs

**FIGURE 1:**
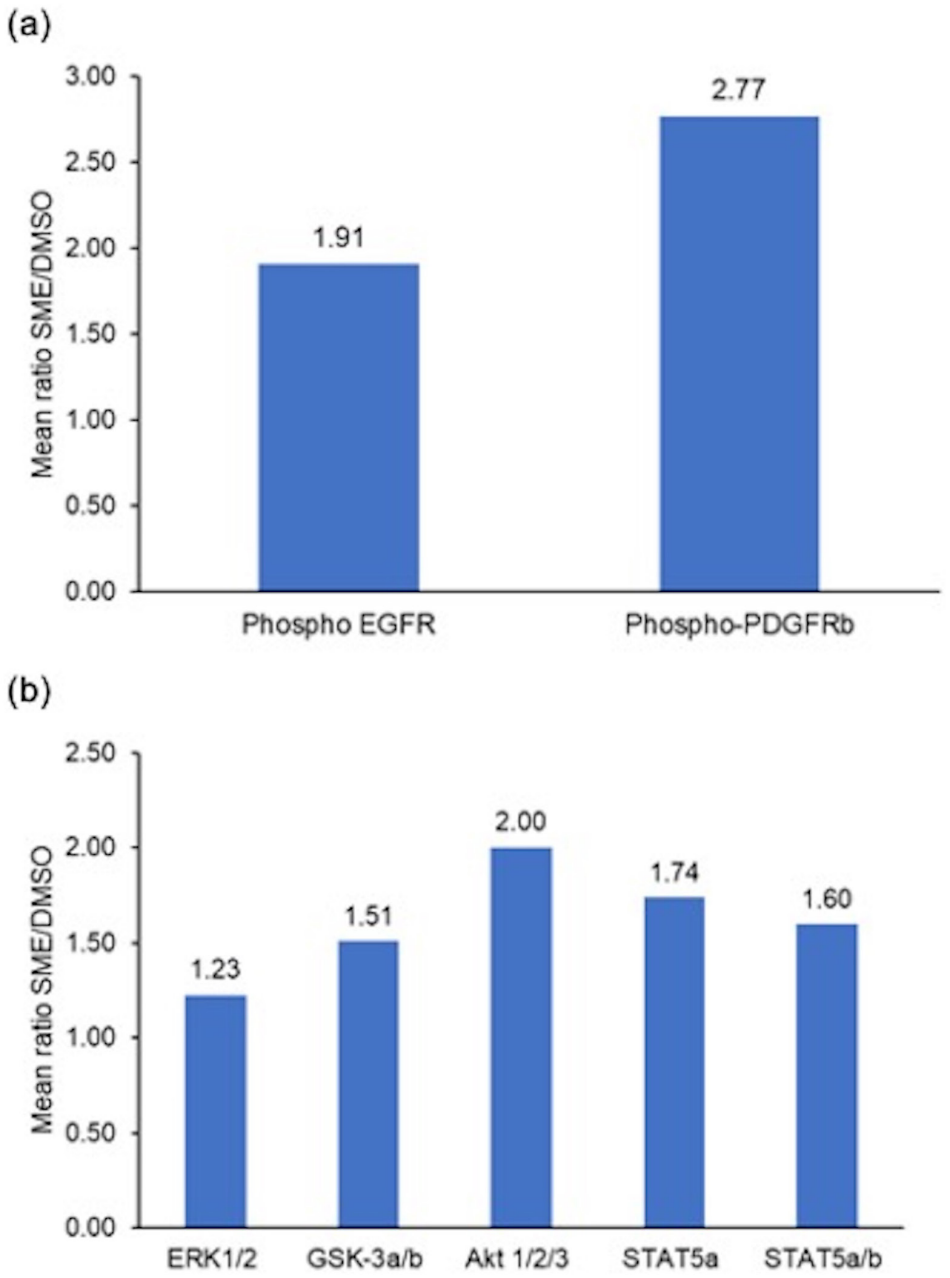
Phosphorylation of receptor tyrosine kinases (RTKs) and their downstream effectors in human follicle dermal papilla cells treated with a *Silybum marianum* extract (SME, 30 μg/mL, dissolved in dimethylsulfoxide [DMSO]) or DMSO for 1 h (*n* = 2 donors, tests performed in duplicate for DMSO‐treated and SME‐treated cells from each donor). (a) EGFR and PDGFRβ, and (b) the downstream phosphokinases ERK1/2, GSK3α/β, Akt1/2/3, STAT5α and STAT5α/β. Data were obtained using the Human Proteome Profiler Phospho‐RTK and Phospho‐Kinase array kits, respectively. Each bar represents the mean ± SEM of SME/DMSO ratio.

EGFR and PDGFR tyrosine phosphorylation levels were higher (1.9‐fold and 2.8‐fold, respectively) in the SME‐treated HFDPCs than in the DMSO‐treated cells (Figure 1a). SME also upregulated phosphorylation of the downstream effectors of these GFRs (i.e., ERK, GSK3, Akt, and STAT5) by 1.2‐ to 2.0‐fold (Figure 1b).

### 3.2 Effect of Manganese PCA on the Wnt/β‐Catenin Pathway and on the Secretion of Versican and VEGF by HFDPCs

**FIGURE 2:**
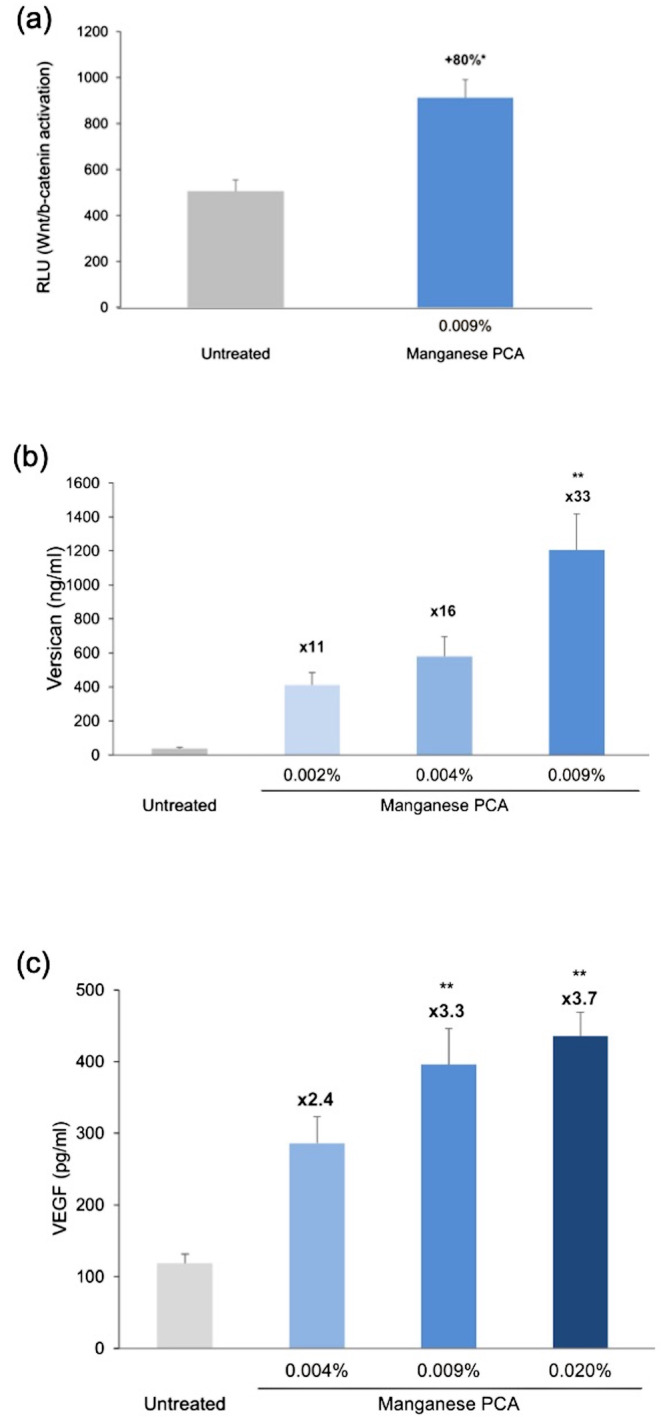
Effects of manganese PCA (MnPCA) on human follicle dermal papilla cells (HFDPCs). (a) Activation of the Wnt/β‐catenin pathway in untreated and MnPCA‐treated HFDPCs. Treated HFDPCs were incubated with 0.009% MnPCA for 24 h. Pathway activation was quantified by measuring the luminescence of HFDPCs transfected with a luciferase reporter gene under the control of a Wnt/β‐catenin promoter (*n* = 3 donors, tests performed in triplicate on untreated and treated cells from each donor). (b, c) The levels of versican and VEGF released into the culture medium of untreated and MnPCA‐treated HFDPCs. Versican and VEGF were quantified by immunoassays, 48 h and 24 h, respectively, after the addition of 0.002%–0.009% MnPCA to the treated HFDPCs (*n* = 3 donors, tests performed in triplicate on untreated and treated cells from each donor). Results show the mean ± SEM of donor triplicates. Percentage and fold increases versus untreated controls are also indicated. *p < 0.05; **p < 0.01 (one‐way ANOVA with a Dunnett's multiple comparison test on data normalized to the untreated controls). RLU, relative luminescence unit.

MnPCA treatment at a dose 0.009% stimulated the Wnt/β‐catenin pathway in the HFDPCs by up to 80% (Figure 2a, *p* < 0.05 vs. untreated controls). MnPCA also significantly enhanced the release of both versican (Figure 2b) and VEGF (Figure 2c) in the HFDPC culture medium in a dose‐dependent manner. Compared to untreated controls, versican, and VEGF secretion were 33.0‐fold higher and 3.3‐fold higher in the 0.009% MnPCA‐treated HFDPCs, respectively (*p* < 0.01 for both comparisons).

### 3.3 Effect of *Lespedeza capitata* Extract on DKK1 Secretion and 5α‐Reductase Activity in HFDPCs

**FIGURE 3:**
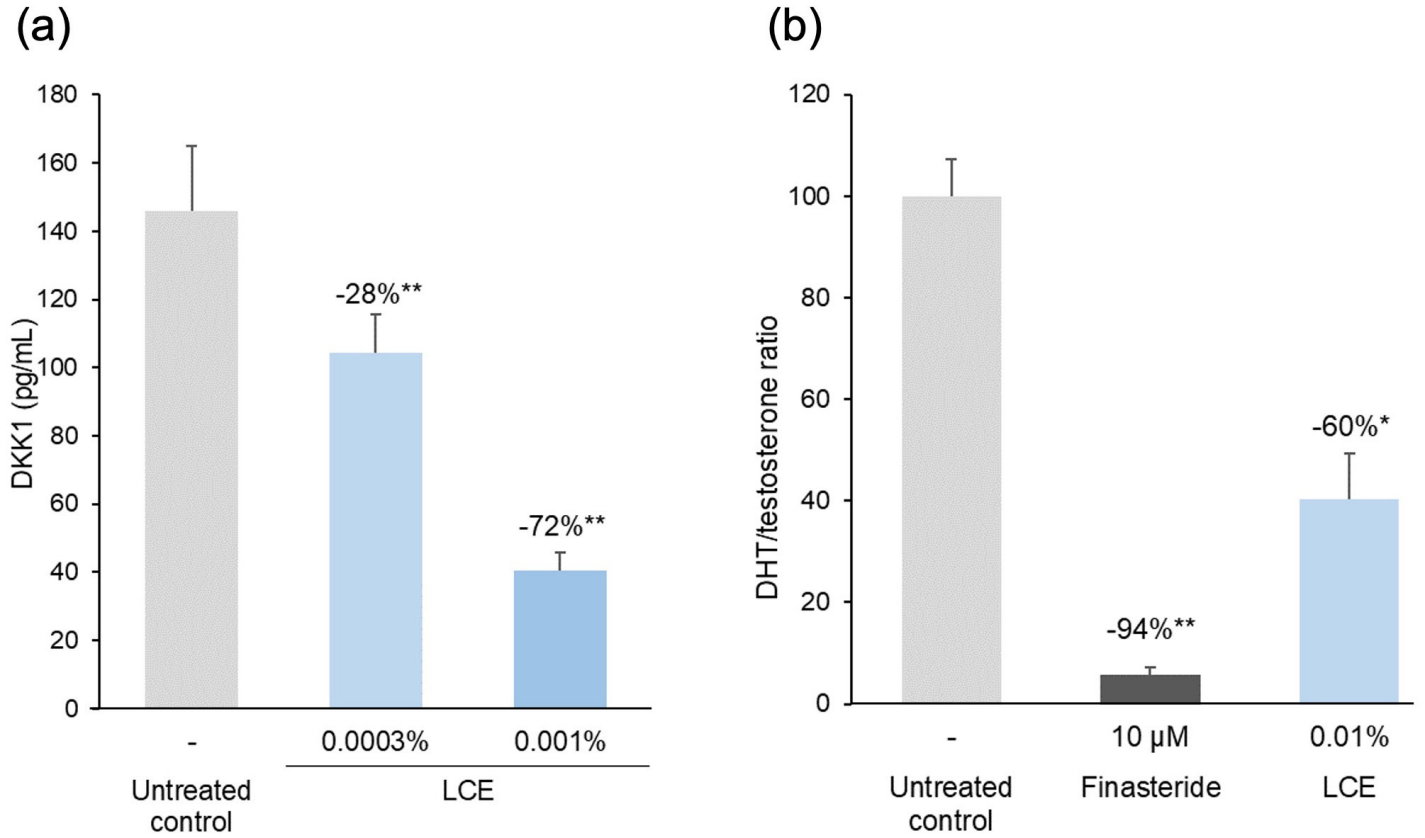
Effect of *Lespedeza capitata* extract (LCE) on Dickkopf‐1 (DDK1) production and 5α‐reductase (5αR) activity in human follicle dermal papilla cells (HFDPCs). (a) Levels of DDK1 in the culture medium of untreated and LCE‐treated HFDPCs. The levels of DDK1 released into the culture medium were quantified by immunoassay 24 h after the addition of LCE to the treated HFDPCs. (b) Dihydrotestosterone (DHT)/testosterone ratio reflecting 5αR activity in untreated, 10 μM finasteride‐treated (positive control) and 0.01% LCE‐treated HFDPCs. The levels of [^14^C]‐DHT and [^14^C]‐testosterone in the culture medium were quantified 24 h after addition of finasteride or LCE to the treated HFDPCs by autoradiography on radioactive thin layer chromatograms. Results show the mean ± SEM of tests performed in triplicate on HFDPCs from three donors (a) and two donors (b); the percentage decreases versus the untreated controls are also indicated. *p < 0.05; **p < 0.01 (one‐way ANOVA with a Dunnett's multiple comparison test on data normalized to the untreated controls).

LCE reduced DKK1 secretion from the HFDPCs in a dose‐dependent manner (Figure 3a). DKK1 levels in the culture medium were 72% lower in 0.001% LCE‐treated HDFPCs than in untreated cells (*p* < 0.01). LCE also appeared to inhibit 5αR activity, as evidenced by the lower DHT/testosterone ratio (−59.6%) in 0.01% LCE‐treated HDFPCs compared to the untreated controls (*p* < 0.05, Figure 3b).

### 3.4 Effect of the Study Serum on Hair Shaft Elongation *Ex Vivo*

**FIGURE 4:**
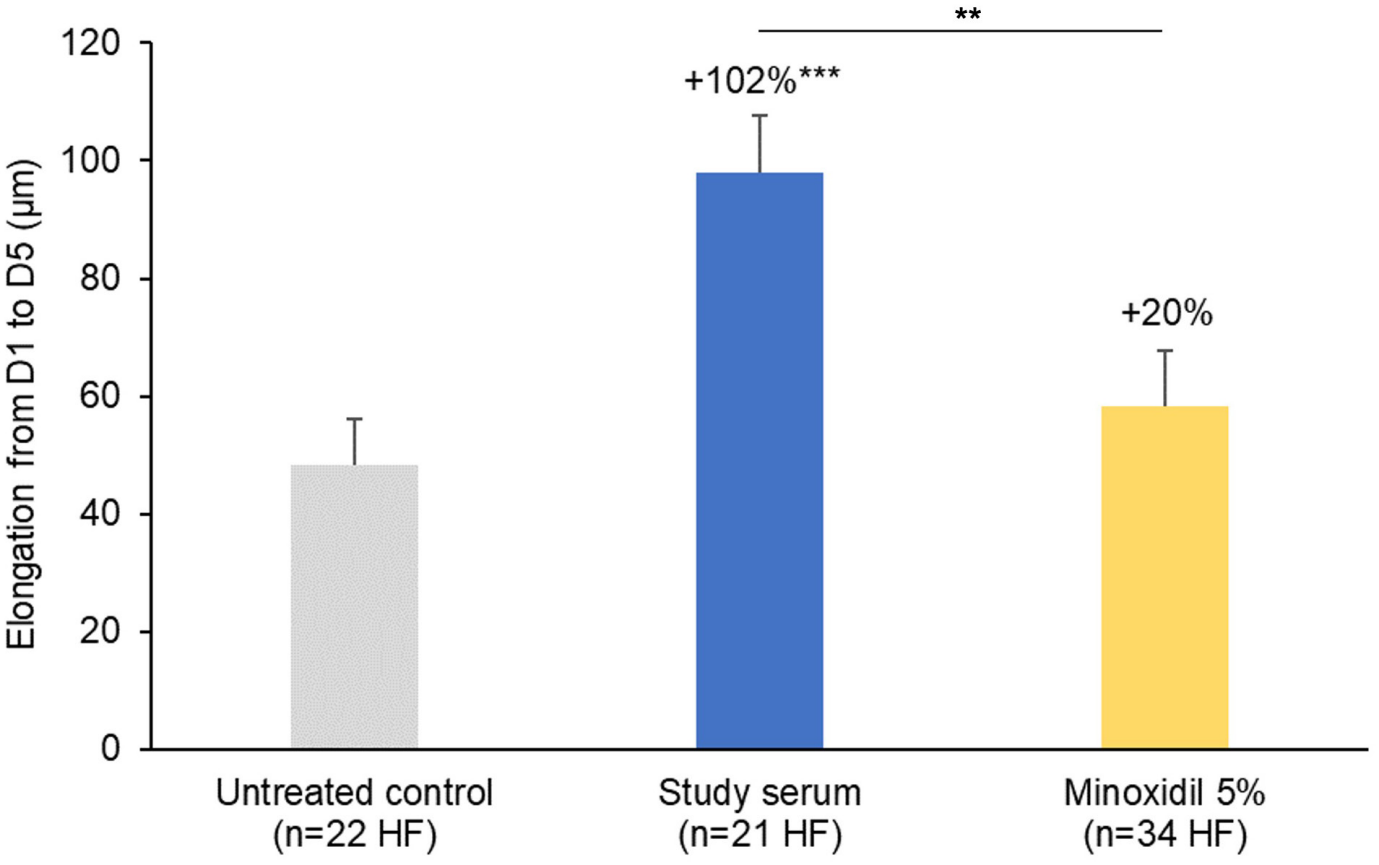
Effect of the study serum on hair shaft elongation after 5 days of *ex vivo* culture of 6 mm‐scalp skin samples in a minimal medium ± topical treatment applied daily from Day 1 to Day 4. Each bar represents the mean ± SEM of the change in length of the hair follicles (HFs) from Day 1 to Day 5 (*n* = 21–34 HFs) from duplicate cultures from two donors. Percentage increases versus untreated controls are also indicated. **p < 0.01; ***p < 0.001 (unpaired *t*‐test).

Elongation of the hair shaft was 2.0‐fold greater in scalp skin samples treated 4 days with the study serum than in untreated controls (*p* < 0.001; Figure 4). Hair shaft elongation in the serum‐treated samples was also significantly greater than that measured after topical treatment with the minoxidil lotion (between‐treatment difference in elongation: 1.7‐fold, *p* < 0.01).

### 3.5 Effect of the Study Serum on the Hair Cycle *Ex Vivo*

**FIGURE 5:**
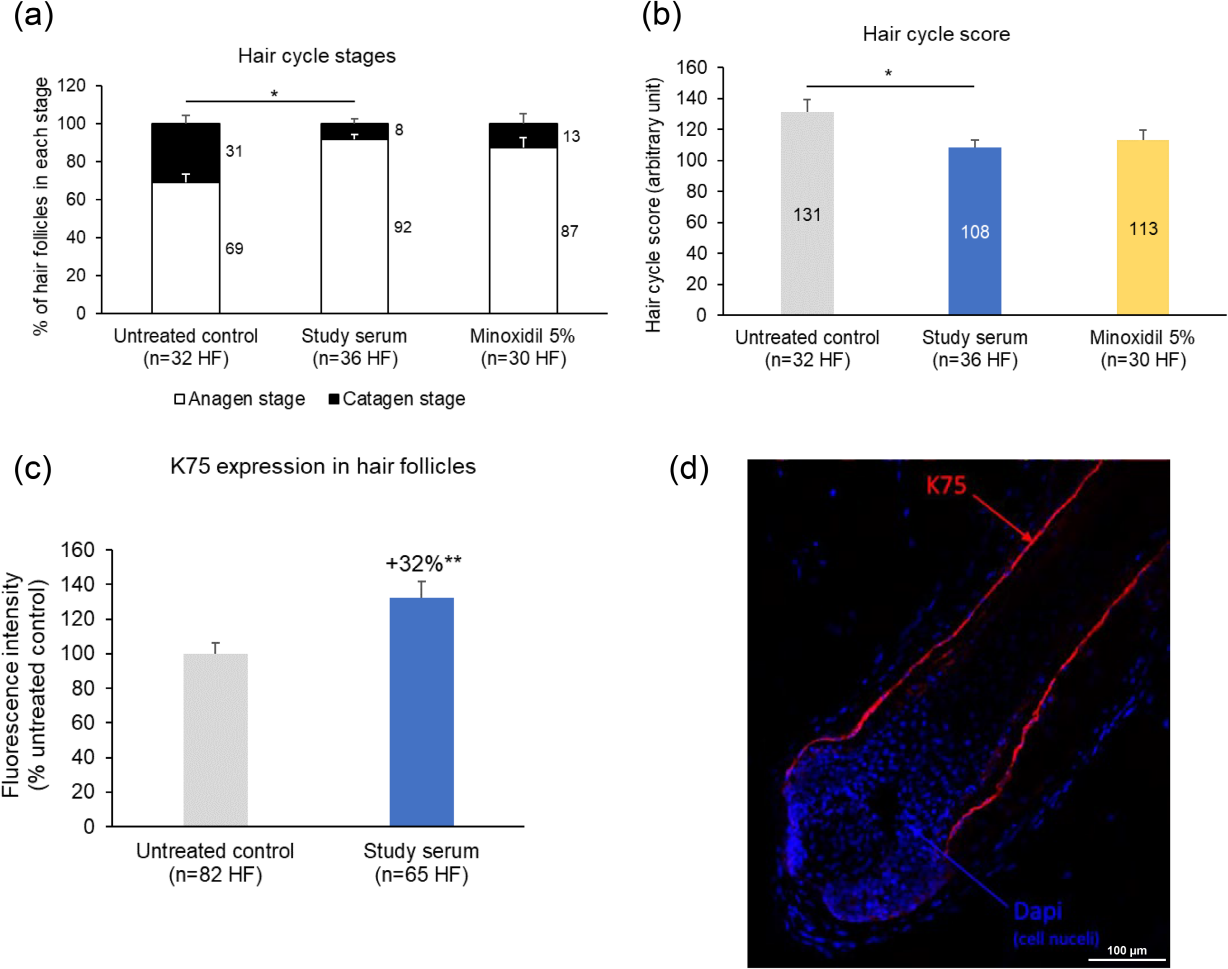
(a) Hair cycle stages, (b) associated hair cycle scores [35], and (c, d) keratin 75 (K75) expression in hair follicles (HFs) after 5 days of *ex vivo* culture of 6‐mm scalp skin samples in a minimal medium ± topical treatment applied daily from Day 1 to Day 4. (a, b, c) Each bar represents the mean ± SEM of analyses performed on duplicate cultures from samples obtained from two donors, and the percentage increase versus the untreated control is also indicated; (a, b) *n* = 30–36 HFs and (c) *n* = 65–82 HFs. (d) Representative confocal microscopy image of K75 immunostaining (in red) and DAPI counterstaining (in blue) of a HF from one donor. *p < 0.05; **p < 0.01 (unpaired *t*‐test).

Among the HFs in anagen on Day 5, only 12% of the untreated HFs and 3% of the study serum‐treated HFs were in the dystrophic anagen phase. None of the HFs were in the mid or late catagen phases. Thus, only HFs in the anagen and early catagen phases were considered for the hair cycle score calculation. At Day 5, the percentage of HFs in the anagen stage was 23% higher in the study serum‐treated cultures than in the untreated cultures (Figure 5a, *p* < 0.05). Hair cycle scores also indicated that more HFs were in the growth phase in the study serum‐treated cultures (Figure 5b, *p* < 0.05 vs. the untreated cultures). No differences were observed between the study serum‐treated and the minoxidil‐treated cultures. The expression of K75 was 32% higher in the cultures treated with the study serum (Figure 5c, *p* < 0.01 vs. the untreated cultures).

### 3.6 Effect of the Study Serum on Hair Matrix Keratinocyte Proliferation and Apoptosis *Ex Vivo*

**FIGURE 6:**
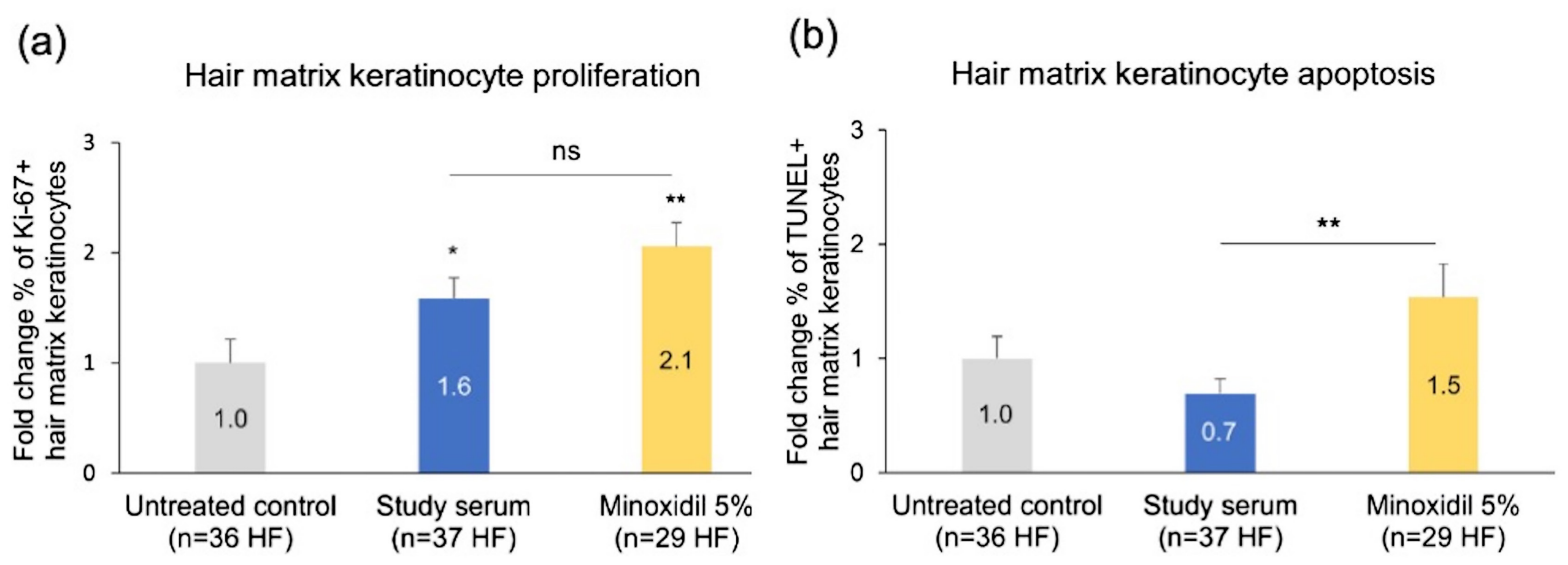
Hair matrix keratinocyte proliferation and apoptosis in untreated and treated *ex vivo* skin samples cultures. (a) Hair matrix keratinocyte proliferation assessed by quantifying the percentage of Ki‐67 + keratinocytes. (b) Hair matrix keratinocyte apoptosis assessed by quantifying the percentage of TUNEL+ keratinocytes. *Ex vivo* cultures of 6‐mm scalp skin samples were grown in a minimal medium ± topical treatment applied daily from Day 1 to Day 4. Data shown are the mean ± SEM fold changes in the percentages of Ki‐67+ or TUNEL+ keratinocytes in the treated cultures relative to the untreated cultures at Day 5, with data for the untreated cultures normalized to 1. Cultures were obtained from two donors and all tests were performed in duplicate. ns, not significant; *p < 0.05; **p < 0.01 (unpaired *t*‐test).

After 5 days of treatment, both the study serum and minoxidil led to significant stimulation of hair matrix keratinocyte proliferation compared to the untreated control (Figure 6a). There were no significant differences in the fold change in percentage of apoptotic cells between the treated and untreated skin sample cell cultures, although the 0.7‐fold change observed for the study serum‐treated samples was significantly smaller than that observed for those treated with minoxidil (1.5‐fold, Figure 6b).

## 4 Discussion

This study assessed the potential beneficial effects of three natural compounds on hair growth using relevant *in vitro* and *ex vivo* human hair models. *In vitro* studies comparing untreated and treated HFDPCs showed that the new patented SME (WO/2021/023820) upregulated the phosphorylation of two growth factor receptors (EGFR and PDGFR) and their downstream effectors (ERK, GSK3, Akt, and STAT). In addition, MnPCA was shown to stimulate the Wnt/β‐catenin pathway and increase versican and VEGF secretion, and the recently patented LCE (*WO/2020/020791A1*) was found to reduce DKK1 secretion and 5αR activity. *Ex vivo* studies comparing treated and untreated scalp skin biopsies showed that a serum containing these three ingredients significantly enhanced hair shaft elongation and prolonged the anagen phase, as demonstrated by an improvement in the hair cycle score. The study serum also promoted hair matrix keratinocyte proliferation, and stimulated K75 expression in the companion layer of the outer root sheath of the HFs.

Hair loss is linked to HF cycle dysfunction, often involving shortening of the anagen phase. PDGF isoforms have been shown to induce and maintain the anagen phase in murine HFs [14]. Moreover, PDGF signals produced by immature intradermal adipocyte lineage cells have been implicated in the control of HF stem cell activation and hair regeneration, and the PDGFR has been shown to be activated during anagen induction in the dermal papilla and the lower part of the hair germ [15]. These findings, together with those of other studies, led Jeong et al. [16] to propose a model for PDGF‐mediated hair growth in which PDGF from HFs and subcutaneous adipocytes binds to the PDGFR of dermal papilla cells and induces the secretion of epiregulin, which in turn binds to the EGFR on follicular keratinocytes, thereby enhancing the proliferation and differentiation of HF K75+ keratinocytes and leading to hair growth. In relation to the findings of current study, our results suggest that SME stimulates hair growth via a molecular mechanism that depends on crosstalk between PDGFR and EGFR signaling in the HF, especially as SME has previously been shown to stimulate K75 expression (patent WO/2021/023820). SME was also found to upregulate the phosphorylation of downstream effectors of EGFR and PDGFR, including Akt. Silibinin, a secondary metabolite of *S. marianum*, has also been shown to activate the Akt signaling pathway in three‐dimensional (3D) cultured human dermal papilla spheroids [37].

The Wnt/β‐catenin signaling pathway plays a key role in stimulating hair growth [9, 10]. Many natural and synthetic compounds that activate the Wnt/β‐catenin signaling pathway have been identified as potential candidates for developing therapies for hair loss, for example, valproic acid; hair stimulating complex containing Wnt7a protein, epidermal growth factors and follistatin; SM04554 [22] as well as tocotrienol (a vitamin E analog); red ginseng oil and its constituents linoleic acid and sitosterol; several terpenoid compounds; several naturally occurring flavonoids and chalcones; ginkolides; watercress and other plant extracts on which light has been shed in the reviews by Choi et al. [19] and Shin et al. [38]. Methyl vanillate has also been shown to promote WNT10B mRNA expression in scalp biopsies of females with AGA, and topical application of this safe plant‐derived ingredient increased hair count and hair mass index in females with AGA [39]. In dermal papilla cells, this pathway has been shown to lead to the production of secreted factors (hepatocyte growth factor [HGF] and insulin‐like growth factor‐1 [IGF1]) that are essential for anagen onset and hair growth, by promoting the proliferation and differentiation of epithelial stem cells in the bulge (anagen onset) and matrix cells (anagen maintenance) [9, 10]. The results of our study showed that MnPCA activated the Wnt/β‐catenin signaling pathway in HFDPCs, providing a potential mechanism by which this active ingredient could promote anagen onset and participate in hair growth. Moreover, we showed that MnPCA enhanced the secretion of versican, a large chondroitin sulfate proteoglycan, in a dose‐dependent manner. The expression of the gene encoding versican is regulated by the β‐catenin signaling pathway. In the dermal papilla, versican expression has been shown to be upregulated during the anagen phase, and downregulated at catagen onset, suggesting that this proteoglycan plays an important role in maintaining the normal growth phase of HFs [12, 13].

A previous study demonstrated that HFDPCs produce VEGF [40]. In the present study, MnPCA was found to enhance the secretion of VEGF by HFDPCs in a dose‐dependent manner. VEGF is an endothelial cell growth factor that promotes angiogenesis and vascular permeability, and thus enhances the supply of oxygen, and nutrients to the HF [41]. Like versican, VEGF expression has been shown to vary during the hair growth cycle: VEGF is widely produced in the anagen phase but its expression decreases when HFs enter the catagen phase. Thus, in addition to promoting anagen onset and hair growth by activating the Wnt/β‐catenin signaling pathway, MnPCA may also prolong the anagen phase by stimulating the extracellular matrix and enhancing the supply of the nutrients necessary for the hair life cycle via its action on VEGF.

Our findings indicate that the third active ingredient tested in our study, LCE, may also improve hair growth by influencing Wnt//β‐catenin signaling. In androgenetic alopecia, Wnt signaling is known to be inhibited through the action of DHT and DKK1 [17, 18, 19, 20, 21]. In this study, we showed that LCE reduced the secretion of DKK1 in a dose‐dependent manner and inhibited 5αR activity, suggesting that this extract modulates Wnt/β‐catenin signaling by inhibiting testosterone metabolism. Further experiments could be performed using a TCF/LEF Luc reporter assay to confirm this hypothesis.

The results obtained from our *in vitro* studies involving the widely used 2D HFDPC culture model therefore provided valuable mechanistic insights into to the potential hair growth promoting effects of the individual test compounds at the cellular level. Indeed, as the signals emitted by the dermal papilla cells play a central role in the regulation of the HF cycle, such studies are important for the identification and development of novel hair growth modulators for cosmetic testing [42]. However, the HF cycle is highly dynamic [5], and involves complex molecular communications between several cell types. *Ex vivo* studies using human scalp skin biopsies are therefore of great interest for further testing [43]. In line with the results obtained *in vitro*, our *ex vivo* studies involving a human scalp skin model showed that the serum containing a combination of SME, MnPCA, and LCE improved hair growth and prolonged the anagen phase compared to untreated controls. Moreover, we found that K75 expression was higher in the companion layer of the outer root sheath of HFs from serum‐treated scalp skin biopsies, most likely due to the action of SME.

The effects of the study serum on the *ex vivo* model were also compared to those of a lotion containing 5% minoxidil, the first‐line treatment for male and female pattern hair loss [3, 22]. Unlike treatment with 5% minoxidil, treatment with the study serum enhanced hair shaft elongation and led to an improvement in the hair cycle score compared to untreated controls. Both treatments stimulated keratinocyte proliferation, and although neither treatment had a significant effect on apoptosis compared to the untreated control, the fold change in the percentage of apoptotic cells in the study serum‐treated skin samples was significantly lower than that for the minoxidil‐treated samples. These data highlight differences in the mode of action of the two treatments. Although minoxidil is a reference treatment for female and male pattern hair loss, this synthetic medicine has been shown to cause side‐effects and to display a subject‐dependent efficacy [2, 3]. There is therefore a need to develop new treatments based on other mechanisms of action. Several herbal ingredients of interest for the treatment of hair loss have been identified in recent years [24, 33, 38, 44, 45, 46, 47, 48]. Our results show that the three ingredients investigated in this study exert promising complementary anti‐hair loss properties in human *in vitro* and *ex vivo* models.

Our study had some limitations. It is difficult to study the longer‐term effects of a topical treatment *ex vivo* because of the limited survival time of cultured scalp biopsies. Moreover, vascularization is important for HF growth: although we showed that MnPCA enhanced VEGF production *in vitro*, the *ex vivo* model used in our study was not vascularized. Lastly, the number of donors was small, but the results obtained *in vitro* and *ex vivo* provided strong evidence of the anti‐hair loss properties of the three ingredients tested. Clinical studies are needed to further test the beneficial effects of these ingredients (see the article by Turlier *et al*. submitted as part of this supplement).

In conclusion, the three tested ingredients displayed complementary anti‐hair loss properties. First, SME modulated cell growth by acting on the EGFR/PDGFR signaling pathways. Second, MnPCA and LCE enhanced the anagen phase by modulating the Wnt/β‐catenin pathway. Moreover, MnPCA also stimulated versican and VEGF production, potentially improving hair anchorage and microcirculation. Finally, LCE reduced DHT levels and inhibited androgen metabolism. *Ex vivo*, the serum combining these active ingredients improved hair growth, prolonged the anagen phase, and enhanced the production of K75, which is involved in hair anchorage. Altogether, our data suggest that products containing a combination of SME, MnPCA, and LCE may be beneficial for treating hair loss.

## Author Contributions

Project development: D.B., M.L., C.M., S.C.; study concept, design, implementation, and management: D.B., M.L., C.M., S.C., S.B.‐T., H.D., N.C.R., J.‐H.S.; sample preparation, data acquisition, data collection: M.‐J.H., A.N.; data analysis: D.B., M.L., C.M., S.C., M.‐J.H., A.N.; data interpretation: D.B., M.L., C.M., S.C.; discussion of the results: D.B., M.L., C.M., S.B.‐T., H.D., N.C.R., J.‐H.S.; critical review of the manuscript: D.B., M.L., C.M., V.M., S.B.‐T., H.D., N.C.R., J.‐H.S. All authors reviewed the manuscript and approved the final version.

## Ethics Statement

The authors confirm that the ethical policies of the journal, as noted on the journal's author guidelines page, have been adhered to. Ex vivo experiments were performed according to the Declaration of Helsinki principles. Informed written consent was obtained from the donors of the temporal scalp skin samples discarded from their routine face‐lift surgery, and the study was approved by the local ethics committee (University of Muenster 2015‐602‐f‐S).

## Conflicts of Interest

D.B., M.L., C.M., M.‐J.H., A.N., V.M., S.C., S.B.‐T., H.D., and N.C.R. are employees of Pierre Fabre Dermo‐Cosmétique & Personal Care, France, and as such received salaries during the period when the research described in this manuscript was conducted. D.B., H.D., and J.‐H.S. are co‐authors of patent WO/2021/023820, and M.L. is a co‐author of patent WO/2020/020791, both filed by Pierre Fabre Dermo‐Cosmétique, France. J.‐H.S. has a translational research contract with and has received personal and Institutional Research Fund from, Pierre Fabre Dermo‐Cosmétique & Personal Care.

## Supporting information


Table S1.

## Data Availability

Data underlying the results presented in this paper may be obtained from the corresponding author upon reasonable request.
