# Supplementary material for: New Plant Extracts Exert Complementary Anti‐Hair Loss Properties in Human In Vitro and Ex Vivo Models
Source: J Cosmet Dermatol. 2024 Nov 28;23(Suppl 5):1–11. doi: 10.1111/jocd.16616 (PMC11603400; doi:10.1111/jocd.16616)
Supplement: Supplementary file 1 — Table S1. [file JOCD-23--s001.docx]

**Table S1** Criteria applied for microscopic hair cycle staging according to Oh et al.^1^

|  | **Hair cycle stage** | | |
| --- | --- | --- | --- |
|  | **Anagen** | **Early catagen** | **Dystrophic (anagen/catagen)** |
| **Dermal papilla** | Large, onion-shaped | Condensed, almond-shaped | As in anagen or catagen |
| **Hair bulb** | Thick matrix envelops DP; located deep in the adipose tissue | Matrix volume loss | Swollen, separation of the epithelial from the mesenchymal tissue |
| **Outer root sheath** | Thick, mutli-layered | As in Anagen | Spongiosis in the ORS |
| **Pigmentation** | HF shaft is mature and pigmented | Melanin incontinence in DP, loss of melanin in precortex and proximal shaft | Appearance of melanin clumping |
| **Hair shaft/follicle length** | Tip of hair shaft emerges through epidermis | Total HF length is not changed | As in anagen or catagen |
| **Hair matrix proliferation** | Prominent proliferating matrix below Auber's line | Decrease in the number of proliferating cells below Auber's line | As in anagen or catagen |
| **Hair matrix apoptosis** | No apoptotic cells in matrix | Low levels of apoptosis may be observed in the hair matrix | As in anagen or catagen, potentially more apoptosis in the connective tissue sheath |

**Abbreviations:** DP, dermal papilla; HF, hair follicle; ORS, outer root sheath

^1^ Oh JW, Kloepper J, Langan EA, et al. A Guide to Studying Human Hair Follicle Cycling In Vivo. J Invest Dermatol. 2016;136(1):34-44
